# First 1000 Days and Beyond After Birth: Gut Microbiota and Necrotizing Enterocolitis in Preterm Infants

**DOI:** 10.3389/fmicb.2022.905380

**Published:** 2022-06-21

**Authors:** Shuqin Zeng, Junjie Ying, Shiping Li, Yi Qu, Dezhi Mu, Shaopu Wang

**Affiliations:** Department of Pediatrics, West China Second University Hospital, Key Laboratory of Birth Defects and Related Diseases of Women and Children (Sichuan University), Ministry of Education, Sichuan University, Chengdu, China

**Keywords:** preterm, gut, microbiota, NEC, infant

## Abstract

Preterm birth remains a major maternal and infant health issue worldwide particularly with an increase in the global preterm birth rate, which requires more interventions to manage the consequences of preterm birth. In addition to traditional complications, recent studies have shown that the succession of gut microbiota of preterm infants is disordered due to the systemic physiological immaturity, which confers negative influences on the growth, development, and health of infants. In the present study, we briefly discussed the prevalence of preterm birth worldwide and then highlighted the signatures of gut microbiota in preterm infants within the first 1000 days of life after the birth categorized into birth, infancy, and childhood. Afterward, we focused on the potential association of clinical phenotypes typically associated with preterm birth (i.e., necrotizing enterocolitis) with gut microbiota, and the potential directions for future studies in this field are finally discussed.

## Introduction

Preterm birth according to the definition of the World Health Organization (WHO) refers to live birth of less than 37 completed gestational weeks counting from the first date of the last menstrual period. Globally, preterm birth still remains a major challenge for maternal and infant health even though advanced developments in medicine and therapeutics have been made in the past years. Causes of preterm birth are multiple, such as infections, inflammations, and maternal stress; however, the precise underlying mechanisms have not been completely understood in most cases (Goldenberg et al., 2008; Kovler et al., 2021; Weitkamp, 2021). Over the short- and long-term course since birth, survived preterm infants face an increased risk of morbidities, such as necrotizing enterocolitis (NEC), sepsis, feeding difficulties, and the dysbiosis of gut microbiota (Chawanpaiboon et al., 2019; Olm et al., 2019).

Diverse microbial communities (i.e., the microbiota) have been characterized and mined at various taxonomic and functional levels across human body sites, such as gastrointestinal tract, oral cavity, skin, breast milk, and vagina (Hill et al., 2017; Ferretti et al., 2018; Fehr et al., 2020). Mounting evidence indicate the linkage between the gut microbiota and human health (Tamburini et al., 2016; Wang et al., 2020a). Notably, the early-life gut microbiota has a long-lasting effect on the development of gut microbiota throughout the life (Roswall et al., 2021), and the dysbiosis of early-life gut microbiota is implicated in changes in the gut microbial trajectory and in an increased risk of chronic diseases, such as diabetes, obesity, and asthma later in life (Keag et al., 2018; Robertson et al., 2019; Wang et al., 2020a).

Preterm infants have been shown to possess differential profiles of gut microbiota, which has drawn great attentions not only from pediatricians but also from microbiologists and immunologists (Hill et al., 2017; Olm et al., 2019; Healy et al., 2022). It is known that the mutualistic relationship between gut microbiota and the immune system in early life is critical for infants’ growth (Gomez de Agüero et al., 2016), and a disturbance of this balance may lead to the occurrence of diseases, in particular NEC (Olm et al., 2019; Healy et al., 2022). In this review, we mainly summarize the current prevalence of preterm birth and changes in gut microbiota in the first 1000 days after birth, then discuss the potential role of gut microbiota in the occurrence of NEC during early life, and finally highlight the future perspective for the microbiota research in preterm birth.

## The Global Prevalence of Preterm Birth

Preterm birth is still a crucial health issue worldwide (Goldenberg et al., 2008; Walani, 2020). Knowing exactly the burden and trend of preterm birth worldwide remains a challenging issue due to the sparsity and incompleteness of the actual records in many countries, which, however, is needed in order to provide more precise interventions from the policies and programs to research and medical treatments (Vogel et al., 2018; Chawanpaiboon et al., 2019). Based on the threshold of fewer than 37 completed weeks of gestation, a recent systematic review of global, regional, and national rates of preterm birth in 2014 was conducted by retrieving the data from 107 countries (Chawanpaiboon et al., 2019). The results indicated that the rate of globally estimated preterm birth was 10.6% with an amount of 14.84 million preterm births. Among the included countries, the highest preterm rate was 13.4% in North Africa, and the lowest preterm rate was 8.7% in Europe. The majority of preterm births (81.1%) occurred in Asia and Sub-Saharan Africa, the middle- and low-income countries. Globally, the preterm birth rate increased from 9.8% in 2000 to 10.6% in 2014 (Chawanpaiboon et al., 2019).

Specific cautions, however, are warranted for interpretation as the systematical search and analysis of data from the publicly available preterm reports may introduce slight biases due to the disproportionate data and several methodological differences across countries, such as the fact that the way and how a preterm birth is monitored and reported (Vogel et al., 2018). The method used to determine the gestational age of a pregnant woman, such as early pregnancy ultrasound (the gold standard for gestational age) and last menstrual period (used in the case of limited access to early pregnancy ultrasound), can result in the incomparable estimation of preterm birth. Other factors include, but are not limited to, the definition of fetal viability, local clinical protocols, data collection, and report strategies, particularly in low- and middle-income countries (Vogel et al., 2018; Chawanpaiboon et al., 2019). For example, Yang and colleagues observed that the preterm birth rate was underestimated to be 7.6% based on the last menstrual period with, which was lower by 1.5% than that based on early ultrasound (Yang et al., 2002). Apart from the actual survey, building a model to predict the rate of preterm birth has also been used as national reports in countries from Asian (including China) and African countries when the availability of data on preterm birth is limited in a national civil registration (Blencowe et al., 2012; Chawanpaiboon et al., 2019). More recently, by establishing a standardized nationwide monitoring system (China’s National Maternal Near Miss Surveillance System) in China, a report regarding the national preterm birth rate in China between 2012 and 2018 was released, which also suggested an increase in the overall rate of preterm births for both singleton and multiple pregnancies, from 5.9% in 2012 to 6.4% in 2018 of all pregnancies (Deng et al., 2021). Indeed, further research is needed nationally and globally to improve our appreciation of the epidemiology of preterm birth, which can guide us to better predict and prevent preterm birth.

## Development of Gut Microbiota in Preterm Birth

**FIGURE 1:**
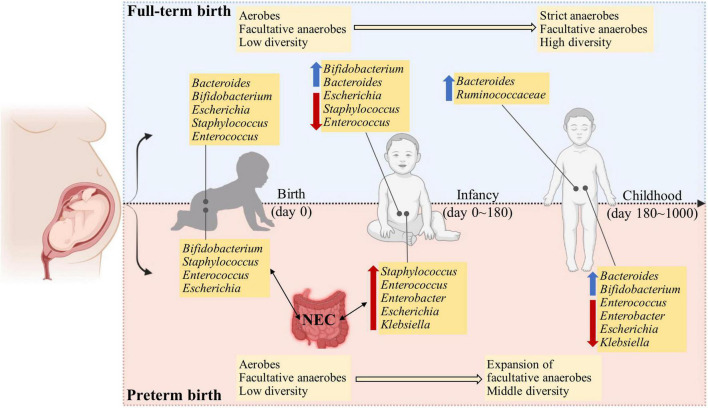
The developmental trajectory and comparisons of full-term and preterm infants in the first 1000 days after birth. Compared to full-term infants, the preterm infants show a dysbiosis of gut microbiota, typically with higher abundance and longer persistence of facultative anaerobes and opportunistic pathogens in early life. This may be attributed to the physiological immaturity and an increase in the oxygen level in the gut lumen, which inhibits the proliferation and colonization of strict anaerobic microbes.

Gut microbiota has been indicated with mounting evidence to be involved in human health and a range of diseases (Tamburini et al., 2016; Ruuskanen et al., 2021). Once delivered, the gastrointestinal tract of infants experience successive waves of microbial exposure and colonization (Bäckhed et al., 2015; Hill et al., 2017; Shao et al., 2019). The origin and determinant of early-life microbial pioneers are not completely understood. The early-life gut microbiota evolves from a relatively simple diversity to gradual maturation toward an adult-like human gut microbiota, which exerts a long-lasting effect in shaping the composition and function of the microbiota throughout life. Meanwhile, the early-life gut microbiota is temporally dynamic, which can be influenced by a number of factors, such as delivery mode, feeding practices, and gestational period (Shao et al., 2019; Fehr et al., 2020; Healy et al., 2022), although to what extent of contribution per factor is still questionable. Compared to full-term infants, preterm infants experience many internal and external challenges, such as physiology, medical treatments, diets, and environments, all of which can detrimentally change the acquisition and development of gut microbiota in early life (Hill et al., 2017; Fouhy et al., 2019). In general, the marked microbial signature of preterm infants in contrast to full-term infants is that most preterm infants are mainly seeded with skin- and hospital-associated microbiota instead of those derived maternally (Rao et al., 2021). Moreover, the gut microbiota of preterm infants is at risk of delayed and altered assembly of their gut microbiota, which may be related to the prolonged presence of facultative anaerobes rather than strict anaerobes (Drell et al., 2014; Ho et al., 2018; Shao et al., 2019; Rao et al., 2021; Figure 1).

### Meconium: Birth

Although the idea of whether the prenatal environment is sterile remains questionable (Fricke and Ravel, 2021), the presence of microbiota has been detected in meconium by using either next-generation sequencing (NGS) or real-time PCR (Morais et al., 2020; Hiltunen et al., 2021; Klopp et al., 2022). The meconium microbiota normally exhibits large between-individual variation, which is influenced by gestational age at birth (Klopp et al., 2022). At the phylum level, the complexity of meconium microbiota is low in most cases with a single phylum, such as Firmicutes, Bacteroidetes, and Proteobacteria, as the most predominant ones (Gómez et al., 2017; Hiltunen et al., 2021; Klopp et al., 2022). The *Bifidobacterium*, *Staphylococcus*, *Enterococcus*, *Streptococcus*, and *Escherichia* are the most abundant genera (Klopp et al., 2022), and these are comparable to full-term infants except for the genus *Bacteroides* which is present with high abundance in full-term infants (Yassour et al., 2018; Wang et al., 2021). This highlights the differences in colonization stability of early microbes between infants born full-term and preterm. When comparing the overall diversity of meconium microbiota from preterm birth to full-term infants, the preterm infants exhibited lower alpha diversity and could be clustered separately based on unweighted UniFrac distance (Hiltunen et al., 2021). The genera of *Bacillus* and *Megamonas* were significantly associated with infants born less than 32 weeks of gestation, and genera of *Proteus* and *Paraprevotella* were significantly associated with infants born from 32 to 37 weeks of gestation (Hiltunen et al., 2021).

Importantly, when transplanting gut microbiota of meconium from infants born preterm to germ-free mice, compared to the mice receiving gut microbiota from full-term infants, the mice showed lower weight gain, increased inflammatory cytokine gene expression, and decreased metabolic hormone levels, suggesting impeded growth, intestinal immune function, and metabolism of the mice receiving gut microbiota from preterm infants (Hiltunen et al., 2021). Notably, mice receiving gut microbiota from infants born from 32 to 37 weeks of gestation exhibited intermediate performance when compared to mice receiving gut microbiota from infants born less than 32 weeks of gestation and those receiving gut microbiota from full-term infants. This phenomenon strongly suggested that the succession of gut microbiota was associated with the gestational age at birth in terms of taxa and microbial functions. However, the detailed responsible elements such as specific microbes, functional genes, and strain-level adaptations warrant further investigation. In addition, whether the profile of microbiota from the meconium is related to the occurrence of neonatal diseases and making early predictions are imperative to explore in the future (Dornelles et al., 2020).

### Days 1–180: Infancy (First 6 Months of Life)

The richness and diversity of gut microbiota are increased as preterm infants grow (Drell et al., 2014), which, however, is lower than the age-matched full-term infants (Arboleya et al., 2012). Compared to full-term infants, that are normally rich with increased beneficial microbes, such as *Bifidobacteria* and *Bacteroides* and decreased facultative microbes when growing, the gut microbiota of preterm infants conversely is characterized with the persistence of Proteobacteria, such as *Klebsiella* and *Escherichia*, and delayed beneficial microbiota as discussed in detail in an earlier study (Healy et al., 2022; Figure 1). In the first week of life, the gut microbiota of preterm infants was dominated by facultative anaerobic bacteria such as mainly *Staphylococcus*, *Enterobacteriaceae*, and *Enterococcus*, while the life was short for the genera of *Bacteroides*, *Bifidobacterium*, and *Lactobacillus* that function as beneficial microbes with resistance against pathogens (Drell et al., 2014). Nevertheless, Drell and colleagues extended the analyses to the first 2 months of life and found no beneficial bacteria as mentioned above, except increases in some facultative anaerobic bacteria from *Klebsiella*, *Veillonella*, *Enterococcus*, and *Escherichia*/*Shigella* (Drell et al., 2014). It has to be mentioned that this pattern of gut microbiota may be associated with a high risk of infection due to microbial translocation from the gut. However, in another study conducted by Hill et al. (2017), the presence of *Bifidobacterium* and *Bacteroides* was detected in preterm infants with gradual increases from the 1st week to 6 months of life. These differences may be attributable to differences in the gestational age, geographical location, and feeding patterns that have been indicated to influence the composition of gut microbiota (Vatanen et al., 2016; Korpela et al., 2018; Fehr et al., 2020). In addition, the gut microbiota has been related to host genetics (Sanna et al., 2022), which, however, is largely relied on adult cohorts, and to our knowledge, influences of host genetics on early-life gut microbiome remain obscure. With a frequent sampling of fecal samples at different intervals from preterm infants until discharge from the hospitals up to 2 months postnatal age, the succession of gut microbiota in preterm infants was indicated to proceed in 4 phases, dominated successively by *Staphylococcus*, *Enterococcus*, *Enterobacter*, and finally *Bifidobacterium* (Korpela et al., 2018). With metagenomic shotgun sequencing, the most prevalent and abundant species across the first 6 months included *Staphylococcus epidermidis*, *Enterococcus faecalis*, *Klebsiella oxytoca*, *Klebsiella pneumoniae*, *Escherichia coli*, and *Enterobacter cloacae* (Gibson et al., 2016).

At a taxonomic rank above the genus, a dichotomous developmental pattern of gut microbiota in preterm infants was observed based on the relative abundance of class Gammaproteobacteria. One group of preterm infants had a low relative abundance of Gammaproteobacteria in the first 2 weeks, which then increased as a function of postnatal age by the 4th week, whereas the second group of preterm infants began with a high relative abundance of Gammaproteobacteria with a gradual decrease by the 3rd week of life. All preterm infants showed a comparable abundance of Gammaproteobacteria by the 4th week of life (Ho et al., 2018). The high relative abundance of Gammaproteobacteria in the second group was highly posited to be vertically transmitted from maternal gut microbiota as vaginal birth was identified to be the leading determinant. However, this hypothesis was not investigated due to the unavailability of maternal fecal samples (Ho et al., 2018). Additionally, the dynamics of classes *Bacilli* and *Clostridia* differed between preterm infants and was significantly involved in the succession of gut microbiota of preterm birth (La Rosa et al., 2014; Ho et al., 2018).

Although the NGS has comprehensively expanded the microbial diversity in early life, NGS is limited to providing the relative abundance of taxa rather than the absolute amount. In order to overcome this limitation, Rao and colleagues developed a cell-based multi-kingdom spike-in method to quantify the absolute abundances within a given microbiota and applied it to the gut microbiota of preterm infants in the first 7 weeks of life (Rao et al., 2021). The total gut bacterial load in the preterm infant gradually increased as infants aged. Being consistent with the findings of Korpela et al. (2018), the gut microbiota during this period of preterm infants also clustered primarily into 4 distinct states, but with different dominant genera such as *Staphylococcus*, *Klebsiella*, *Escherichia*, or *Enterococcus*, independent of diet or delivery mode. Notably, both the first community states were dominated by the genus *Staphylococcus* in the early stage of life (Korpela et al., 2018; Rao et al., 2021). Following that, the community state gradually shifted to one dominated by *Klebsiella*, *Enterococcus*, or *Escherichia*. Apart from the inconsistency in sampling time points, this may also be caused by other technical factors, for example, the sequencing approach (16S amplicon sequencing vs. shotgun sequencing), and the quantitative method (relative vs. absolute abundance).

### Breast Milk Microbiota of Preterm Birth

Breast milk contains a large range of macronutrients- and micronutrients, and microbes that play a vital role in shaping the gut microbiota of infants affect short-term and long-term health (Ahern et al., 2019; Fehr et al., 2020). Our current knowledge about breast milk microbiota is largely from healthy mothers who gave birth at full-term, which may differ from breast milk produced by the mothers who had a preterm birth while considering complications normally associated with preterm birth and extended mother-infant separation. Encouragingly, Asbury and colleagues characterized the taxonomic profile of breast milk of 86 preterm mothers and found overall changes in the abundance of certain genera over the first 8 postpartum weeks and highly individualized temporal changes (Asbury et al., 2020). The 5 most predominant genera included *Staphylococcus*, *Acinetobacter*, *Pseudomonas*, *Corynebacterium*, and *Streptococcus* over all investigated preterm mother’s milk, which was influenced by maternal characteristics, such as delivery mode, pre-pregnancy BMI, and the class, timing, and duration of antibiotics. This pattern of microbiota was slightly different from the breast milk of full-term mothers, which mainly comprised *Streptococcus*, *Pseudomonas*, and *Staphylococcus* (Murphy et al., 2017; Wang et al., 2020b). Genus *Streptococcus* is commonly present in the oral microbial community and may be one origin of breast milk microbiota, and the decreased abundance of *Streptococcus* in mothers of preterm birth may reflect infants receiving milk mainly by feeding tube during their hospital course (Asbury et al., 2020). More research on the composition and succession of microbiota from breast milk of mothers giving preterm birth is needed, which is supposed to be much complicated than that of full-term mothers, due to a longer stay in hospital and clinical treatments.

### Days 180–1000 and Beyond: Childhood

As described above, the gut microbiota of preterm birth in infancy differs from full-term infants, which is proposed to leave a longer-term influence on the gut microbiota later in life. However, the longitudinal follow-up of preterm infants to elucidate the development of gut microbiota toward an adult-like pattern is rarely available.

The increasing microbial diversity and decreasing interindividual variability were observed as preterm infants aged (Gómez et al., 2017). Firmicutes became the most abundant phylum, followed by Actinobacteria and Bacteroidetes at 2 years of age instead of phylum Proteobacteria at 3 weeks of life (Gómez et al., 2017). Notably, the majority of genera that were hospital-associated were decreased, such as *Escherichia coli*, *Enterobacter aerogenes*, *Klebsiella pneumoniae*, and species from *Enterococcus*, *Granulicatella*, *Serratia*, *Proteus*, and *Yersinia*. Consistent with the changes in diets (e.g., introduction of solid food), the abundances of microbes that can degrade the carbohydrates were increased, such as *Bacteroides vulgatus*, *Ruminococcus obeum*, *Ruminococcus bromii*, and *Lactococcus*. Additionally, microbes with the capacity to produce butyrate, such as *Anaerostipes caccae*, *Eubacterium hallii*, and *Coprococcus eutactus*, became prevalent (Gómez et al., 2017). However, compared to full-term infants, preterm infants at 2 years of age still showed a lower abundance of *Bifidobacterium* and *Lactobacillus* (Gómez et al., 2017).

A recent study with fecal sampling of preterm and full-term infants of up to 4 years of age demonstrated that the gut microbiota of preterm infants overall exhibited lower alpha diversity compared to full-term infants and clustered separately at year 1 (Fouhy et al., 2019). The gut microbiota of preterm infants at year 4 was clustered with that of full-term infants at year 2, indicating a delayed succession of gut microbiota of preterm infants (Fouhy et al., 2019). Several discriminatory taxa at different levels associated with gestational age were capable to be detected. In year 1, *Lactobacillus* and *Coprobacillus* showed the greatest discriminatory power for preterm infants, while full-term infants were discriminated by *Bacteroides* and *Fecalibacterium* with the greatest power. In year 2, the discriminative genera for preterm infants included *Streptococcus*, while *Parabacteroides* was associated with full-term infants. In year 4, some discriminative taxa were still detected between preterm and full-term infants, i.e., *Carnobacterium*, *Desulfovibrio*, and *Phascolarctobacterium* for preterm infants and several species of *Christensenellaceae*, *Lachnospiraceae*, and *Ruminococcaceae* for full-term infants (Fouhy et al., 2019).

## Associations of Gut Microbiota With Necrotizing Enterocolitis

The NEC has attracted a lot of attention of researchers from different fields as this disease can progress to bowel necrosis, sepsis, and mortality (Olm et al., 2019). The pathogenesis of NEC remains largely unknown, and biomarkers to identify individuals at high risk of NEC are also lacking. With the advancement of sequencing technologies, NEC is proven to associate with altered gut microbiota, i.e., specific overrepresented or underrepresented species (Aziz et al., 2022; Healy et al., 2022). However, the causal relationship and mechanisms between NEC and altered gut microbiota are not fully determined.

As discussed above, preterm infants normally harbor an increased abundance of *Enterobacteriaceae* compared to full-term infants, and this signature of gut microbiota has been related to the onset of NEC in multiple studies (Morrow et al., 2013; Olm et al., 2019). In order to exactly learn what happens in the period immediately before NEC diagnosis, Olm and colleagues longitudinally collected the stool from preterm infants who developed NEC later (pre-NEC) and unexpectedly found that *Enterobacteriaceae* was not significantly enriched in the gut microbiota of preterm infants who developed NEC (Olm et al., 2019). Based on this observation, the author suspected that the positive association between *Enterobacteriaceae* and NEC may be caused by antibiotic intervention (Olm et al., 2019). At a higher taxonomic rank, *Klebsiella pneumoniae* was found to be the most prevalent in the pre-NEC samples (52%) rather than in control samples (23%) (Olm et al., 2019). Notably, replication rates of all the detected bacteria, particularly *Enterobacteriaceae*, were increased in pre-NEC samples, which may promote disease onset (Olm et al., 2019). Further research indicated that the association of altered gut microbiota with NEC can be stratified by the bacterial capacity of binding maternal IgA (Gopalakrishna et al., 2019). In the study carried out by Gopalakrishna et al. (2019) maternal milk was the predominant source of IgA in the gut in the first month of life, and a decrease in IgA-bound bacteria was attributed to the development of NEC, and *Enterobacteriaceae* was dominated in the IgA-unbound fraction of the bacteria before the onset of NEC. However, due to the limitation of taxonomic resolution from sequencing technology (i.e., 16S amplicon sequencing) in this study, mechanisms underlying the loss of IgA binding capacity remain unexplored, which may be caused by the microbial genomic changes and strain adaptation.

In order to move the microbiota research from associations to mechanisms, different methods have been adopted to build a NEC-like model with animals for a better understanding of the pathogenesis of the disease (Ares et al., 2018). Mihi et al. (2021) with the murine NEC model observed the dynamic expression of IL-22 in the terminal ileum of healthy neonatal mice across the prenatal and postnatal periods, i.e., low-level expression before weaning and increasing afterward. Although the deficiency of IL-22 did not increase the susceptibility of neonatal mice to NEC, the authors found that IL-22 administration reduced NEC severity, enhanced epithelial cell regeneration, and protected the integrity of the intestinal epithelial barrier (Mihi et al., 2021). Surprisingly, IL-22 administration did not affect the diversity and the community composition of gut microbiota in NEC-induced model, providing a potential therapeutic strategy for NEC treatment.

## Conclusion

The prevalence of preterm birth is globally increasing, and associated complications are recognized as the leading cause of early-life mortality, which thus require new interventions to manage the consequences of preterm birth in the future. We understand that early life is a critical window for the development of the immune system and gut microbiota. As discussed above, the gut microbiota of preterm birth lacks beneficial microbes, however, compared to full-term birth, and our understanding of gut microbiota of preterm birth is still limited, in particular, the temporal dynamics across the early life based on longitudinal cohorts and the absolute microbial abundances. Additionally, investigations into interactions between gut microbiota and various diseases in preterm birth, such as NEC and sepsis that are closely related to microbes, are urgently necessary. It is imperative to further disclose a range of specific biomarkers and build a predictive model for diseases. Based on the differential taxa and functions across the health and diseases, rational selection and isolation of efficacious probiotic strains from the gut or other sources can be served as novel strategies for preventing diseases, such as personalized probiotics and artificial microbial consortia. Moreover, breastfeeding has been suggested as an effective strategy to decrease the incidence of NEC; however, the underlying mechanisms such as the effective components of breast milk and the relevant mode of action need to be further explored. All these endeavors will help to predict, prevent, and finally protect infants from diseases in clinical practice.

## Author Contributions

All authors wrote, reviewed, and approved this manuscript for publication.

## Conflict of Interest

The authors declare that the research was conducted in the absence of any commercial or financial relationships that could be construed as a potential conflict of interest.

## Publisher’s Note

All claims expressed in this article are solely those of the authors and do not necessarily represent those of their affiliated organizations, or those of the publisher, the editors and the reviewers. Any product that may be evaluated in this article, or claim that may be made by its manufacturer, is not guaranteed or endorsed by the publisher.
